# Tumor-associated mutations in a conserved structural motif alter physical and biochemical properties of human RAD51 recombinase

**DOI:** 10.1093/nar/gku1337

**Published:** 2014-12-24

**Authors:** Jianhong Chen, Milagros D. Morrical, Katherine A. Donigan, Joanne B. Weidhaas, Joann B. Sweasy, April M. Averill, Jennifer A. Tomczak, Scott W. Morrical

**Affiliations:** 1 Department of Biochemistry, University of Vermont College of Medicine, Burlington, VT 05405, USA; 2 Department of Therapeutic Radiology, Yale University School of Medicine, New Haven, CT 06520, USA; 3 Department of Radiation Oncology, University of California-Los Angeles, Los Angeles, CA 90095, USA; 4 Department of Microbiology and Molecular Genetics, University of Vermont College of Medicine, Burlington, VT 05405, USA

## Abstract

Human RAD51 protein catalyzes DNA pairing and strand exchange reactions that are central to homologous recombination and homology-directed DNA repair. Successful recombination/repair requires the formation of a presynaptic filament of RAD51 on ssDNA. Mutations in BRCA2 and other proteins that control RAD51 activity are associated with human cancer. Here we describe a set of mutations associated with human breast tumors that occur in a common structural motif of RAD51. Tumor-associated D149N, R150Q and G151D mutations map to a Schellman loop motif located on the surface of the RecA homology domain of RAD51. All three variants are proficient in DNA strand exchange, but G151D is slightly more sensitive to salt than wild-type (WT). Both G151D and R150Q exhibit markedly lower catalytic efficiency for adenosine triphosphate hydrolysis compared to WT. All three mutations alter the physical properties of RAD51 nucleoprotein filaments, with G151D showing the most dramatic changes. G151D forms mixed nucleoprotein filaments with WT RAD51 that have intermediate properties compared to unmixed filaments. These findings raise the possibility that mutations in RAD51 itself may contribute to genome instability in tumor cells, either directly through changes in recombinase properties, or indirectly through changes in interactions with regulatory proteins.

## INTRODUCTION

Human RAD51 protein is a RecA-like DNA strand transferase that catalyzes the central reactions of homologous pairing and DNA strand exchange in the homologous recombination (HR) and homology-directed repair (HDR) pathways. Given the crucial roles played by HR in distinctive cellular genome stability processes, a linkage between HR dysfunction and cancer phenotype is now clear and widely accepted (1). The causal relationship between RAD51 abnormalities and cancer, however, is poorly understood.

Elevated expression of RAD51 protein has been observed in a wide variety of cancers suggesting that overexpression of RAD51 confers advantages to tumor cells (2). Increased RAD51 expression was found in invasive ductal breast cancer and the level of overexpression correlated directly with the histological grading of the tumor (3). As HR machinery components have now become crucial targets for cancer treatment, there is a widely observed correlation between expression levels of RAD51 and resistance to therapeutic drugs (4). High RAD51 expression was also reported in malignant prostate and small cell lung cancers (5). Conversely, the expression of RAD51, as well as BRCA1, is reduced in almost one third of breast tumor cell lines and primary sporadic breast cancer cells (6–9). Consistent with the drug resistance of the cancer cells conferred by the overexpression of RAD51, down-regulation of RAD51 also increased the radio-sensitivity of prostate cancer cells (10) and malignant glioma cells (11). Although the mechanism and significance of such deregulation of RAD51 remains speculative, a basal level of RAD51 activity appears to be kept in an elaborate balance to ensure appropriate functionality of RAD51-dependent HR in specific contexts.

Another approach to assess the risk of carcinogenesis associated with RAD51 is to explore naturally occurring *RAD51* polymorphisms across the population. An early study conducted in Japanese hereditary breast cancer patients found 2 out of 45 patients contained a RAD51–R150Q variant that is absent among 200 sporadic breast cancer or 100 colon cancer cases, which suggested this site could be a disease-associated mutation (12). However, the same mutation was missed in a screen conducted in 46 well-characterized *brca1/2*-negative breast cancer families (13). A single-nucleotide variant affecting the 5′-untranslated region of *RAD51* was shown to increase breast cancer risk in carriers of mutant *brca2* alleles but not in non-carriers (14–16). Meanwhile, two different single-nucleotide variants affecting intron regions of *RAD51* were shown to increase cancer risk in carriers of mutations in the *BLM* gene (17). In addition to the BRCA2 and BLM proteins, RAD51 interacts directly or indirectly with other key players in tumorigenesis including p53, PALB2, BRCA1 and Ataxia telangiectasia mutated (ATM) (18). Therefore, it is reasonable to postulate that mutations affecting RAD51 activity or its interactions with other HR/HDR machinery could contribute to genomic instability and the generation of tumorigenic mutations.

Despite extensive studies of human RAD51, there are few reports on the basic biochemical behavior of tumor-associated variants. The molecular properties of RAD51 protein include ssDNA- and dsDNA-binding activities, ssDNA-stimulated adenosine triphosphate (ATP) hydrolysis and ATP-dependent pairing and exchange of homologous ssDNA/dsDNA segments (19). The enzymatic activities of RAD51 are activated upon its assembly into a right-handed, helical presynaptic filament on ssDNA. The RAD51 presynaptic filament is a dynamic nucleoprotein structure that undergoes allosteric transitions in response to ATP binding and hydrolysis (19–21). The ATP-bound form of the filament, considered to be the active form, has a high helical pitch of 90–130Å and contains ssDNA that is significantly stretched (20). The ADP-bound form of the filament, considered to be the inactive form, has a low helical pitch of 65–85Å. In addition to regulation by the ATPase cycle, presynaptic filament dynamics are regulated by mediator proteins including BRCA2 and RAD51 paralogs, by DNA helicases and motor proteins including BLM and RAD54, and likely by signaling proteins and post-translational modifications (19,22,23).

As new genetic determinants of cancer are continually identified, it is striking that many involve mutations affecting proteins that are regulators of RAD51, or even act directly as mediators of RAD51-catalyzed reactions (24,25). Evidence for direct linkages between RAD51 variants and cancer is still limited, however. One limitation is that most RAD51 mutations reported to date are located in the non-coding regions of its gene, due to the low penetrance of RAD51 missense mutations (13). Here we report two novel, breast tumor-associated mutations in the coding sequence of RAD51 protein, D149N and G151D, which map adjacent to the previously described mutation R150Q that was associated with bilateral breast cancer (12). All of these mutations alter RAD51 physical properties, and two of them affect RAD51 catalytic properties *in vitro*. All three of the variants map to a conserved structural motif on the surface of the RAD51 filament that may participate in regulatory interactions with other molecules. The results suggest that mutations in RAD51 protein could play important roles in carcinogenesis and tumor progression.

## MATERIALS AND METHODS

### Reagents

Chemicals, biochemicals and enzymes were purchased from Sigma-Aldrich unless specifically stated. Restriction enzymes and T4 polynucleotide kinase were purchased from New England Biolabs. All reagents were analytical grade and solutions were made with Barnstead NANO-pure water. Tris-EDTA (TE) buffer contained 10 mM Tris–HCl, pH 8.0 and 1 mM ethylenediamine tetraacetic acid (EDTA).

### Nucleic Acids

Oligonucleotides were purchased from Operon. Circular M13mp18 ssDNA (7.3 kb) was prepared as described (26). Circular φX174 ssDNA (5.4 kb) was purchased from New England Biolabs. Supercoiled M13mp18 dsDNA (7.3 kbp) was purchased from New England Biolabs and was linearized by digestion with XbaI restriction endonuclease. Supercoiled φX174 dsDNA (5.4 kbp) was purchased from Promega and was linearized by digestion with PstI restriction endonuclease. Linearized dsDNA molecules were purified by phenol extraction and ethanol precipitation. The concentrations of ssDNA and dsDNA stock solutions were determined according to manufacturer's instructions, and are expressed in units of micromoles of nucleotide residues per liter. All DNA molecules were stored at −20°C in TE buffer.

### Sequencing of genomic DNA

Extraction and sequencing of genomic DNA was carried out as previously described (27). Fresh, frozen breast tumor samples were obtained from Yale-New Haven Hospital. Genomic DNA was purified by QIAmp kit (Qiagen). Exon 6 of the human *RAD51* gene (NM_002875) was amplified by nested polymerase chain reaction (PCR) using the following primers (Table 1): Oligo 7 (forward #1), oligo 8 (reverse #1), oligo 9 (forward #2) and oligo 10 (reverse #2). PCR reactions were carried out using the high-fidelity *Pfu* polymerase for 35 cycles of 97°C for 2 min → 96°C for 30 s → 59.5°C for 30 s → 72°C for 1 min; followed by 72°C for 5 min and 10°C until electrophoresis. Consensus sequences were obtained from the AceView database, Human 2007 build (http://www.ncbi.nlm.nih.gov/IEB/Research/Acembly/index.html). Specificity of primer sequences was confirmed by alignment to the human genome using BLAST (http://blast.ncbi.nlm.nih.gov/Blast.cgi). Direct sequencing of the PCR products was performed at the Keck DNA Sequencing Facility at the Yale University School of Medicine. Chromatogram files were visualized and aligned to the consensus sequence using the Geneious software program (www.geneious.com, Version 3.8.5). Amplification occurred in 32 of 43 total samples (∼75%) and two mutations were identified, heterozygous G151D and homozygous D149N. These mutations were found once each, in separate tumors.

**Table 1.** Lengths and sequences of oligonucleotide primers used for site-directed mutagenesis and DNA amplification

Oligonucleotide	Length (b)	5′ → 3′ Sequence
1	39	ACCTGCCAGCTTCCCATTAACCGGGGTGGAGGTGAAGGA
2	39	TCCTTCACCTCCACCCCGGTTAATGGGAAGCTGGCAGGT
3	38	GCCAGCTTCCCATTGACCAGGGTGGAGGTGAAGGAAAG
4	38	CTTTCCTTCACCTCCACCCTGGTCAATGGGAAGCTGGC
5	39	CAGCTTCCCATTGACCGGGATGGAGGTGAAGGAAAGGCC
6	39	GGCCTTTCCTTCACCTCCATCCCGGTCAATGGGAAGCTG
7	22	CCAGTTCTGACAGGACTAGCTG
8	23	GGACGTGGGGTTATAGAAGAGAC
9	21	GTCATGAGGAGCTTGGTCAGC
10	21	GCCAAACTAACCCTGGCAATC

Oligonucleotides 1–6 were used for QuikChange site-directed mutagenesis of human RAD51. Underlined trinucleotide sequences represent codon changes with respect to wild-type RAD51. Oligonucleotides 7–10 were used for nested PCR to amplify and sequence exon 6 of the *RAD51* gene from breast tumors. See ‘Materials and Methods’ section for details.

### Site-directed mutagenesis of human RAD51 protein

Plasmid pET-15b expressing a His_6_-tagged version of the human RAD51 protein was a generous gift from Dr Hitoshi Kurumizaka at Waseda University, Japan. D149N, R150Q and G151D mutations were introduced separately using the QuikChange site-directed mutagenesis protocol (Stratagene). The primers used are shown in Table 1 (oligos 1 and 2 for D149N; oligos 3 and 4 for R150Q; oligos 5 and 6 for G151D). PCR reactions were carried out according to the QuikChange protocol and the resulting plasmids were sequenced at the Vermont Cancer Center DNA Analysis Facility to verify successful mutagenesis.

### Purification of human RPA and RAD51 proteins

Human Replication Protein A (RPA) expression plasmid p11d-tRPA was a kind gift from Dr Marc S. Wold at the University of Iowa. RPA was expressed in *Escherichia coli* strain BL21 (DE3) and the protein was purified as described (28,29).

Human RAD51 proteins were expressed from plasmid pET-15b harboring wild-type (WT) or mutant versions of the *RAD51* gene, which was transformed into Rosetta2 (DE3) competent cells (Novagen, Inc., Madison, WI). The expression and purification steps were a modified version of a published protocol (30), which was used for purifying both WT and the three mutant RAD51 proteins. Briefly, an auto-induction protocol (31) was used for expressing the His_6_-tagged RAD51 protein in the *E.coli* Rosetta2 (DE3) strain, which also carries an expression vector for the tRNAs (pRARE2, Novagen). Freshly transformed colonies were used to inoculate 20 ml cultures of LB plus 100 μg/ml ampicillin. After overnight growth at 37°C, the cultures were used to inoculate 2 l of auto-induction media consisting of Terrific Broth (USB products), plus 0.5% glycerol, 0.05% glucose, 0.2% α-lactose monohydrate and 100 μg/ml ampicillin. Cells were grown with vigorous shaking at 20°C for 48–60 h and then harvested by centrifugation. Each RAD51 protein was purified from cell lysate by affinity chromatography on a HiTrap chelating column (GE Life Sciences) charged with 100 mM nickel sulfate according to manufacturer's instructions. The His_6_ tag was removed using thrombin protease (GE Healthcare Bio-Sciences). RAD51-containing fractions were chromatographed on a second HiTrap chelating column charged with 100 mM nickel sulfate. RAD51-containing fractions were dialyzed into 50 mM Tris, pH 8.0, 200 mM KCl, 0.25 mM EDTA, 2 mM 2-mercaptoethanol, 10% Glycerol. RAD51 protein was further purified by chromatography on a MonoQ-sepharose column (GE Healthcare Bio-Sciences) (50 mM Tris, pH 8.0, 200 mM KCl, 0.25 mM EDTA, 2 mM 2-mercaptoethanol, 10% Glycerol and a linear salt gradient to 600 mM KCl) to remove a nuclease contaminant. The purified protein was dialyzed into storage buffer consisting of 20 mM HEPES (pH 7.5), 150 mM NaCl, 0.1 mM EDTA, 2 mM 2-mercaptoethanol and 10% glycerol, frozen in liquid nitrogen and stored at −80°C.

Protein concentrations were determined by the Bradford method with bovine serum albumin as the standard. All protein stock solutions were >95% pure and nuclease-free as determined by electrophoretic assays.

### ATPase assays

Steady-state rates of ATP hydrolysis were measured using thin-layer chromatography (TLC) assays. Reactions at 37°C contained 30 mM HEPES (pH 7.5), 1 mM MgCl_2_, 30 mM KCl, 45 mM NaCl, 0.03 mM EDTA, 1 mM DTT, 3% glycerol and 0.1 mg/ml bovine serum albumin (BSA) in a final volume of 50 μl. In addition the reaction mixtures contained 2.0 μM WT or variant RAD51 and varying amounts of M13mp18 ssDNA and ATP (containing 10 μC_i_/ml of α-[^32^P]-ATP) depending on the experiment. Experiments with ATP as the variable substrate used a constant ssDNA concentration of 6 μM, which is stoichiometric with respect to RAD51 concentration assuming a binding site size of 3 nucleotide residues per protomer. Experiments with ssDNA as the variable used a constant ATP concentration of 1 mM. Reactions were initiated by the addition of ATP + α-[^32^P]-ATP. Aliquots (8 μl) were removed at various time points and quenched with 0.1 M EDTA and 1% sodium dodecyl sulphate (SDS) (final concentrations). Quenched samples were spotted out at 1 μl volume onto PEI-cellulose TLC plates (20 × 20 cm) at 1 cm intervals. After all the samples were spotted and dried the TLC plates were developed with 0.75 M KH_2_PO_4_ and allowed to air dry. The TLC plates were exposed for 1.5 h to a K-screen (Kodak) and scanned by a Bio-Rad Personal Molecular Imager-FX (Vermont Cancer Center DNA Analysis Facility). Quantification of the phosphorimage was performed by Quantity One v4.5.1 (Bio-Rad) software and subsequently fit using Graphpad Prism v5.0 (Graphpad Software Inc).

### DNA strand exchange assays

DNA strand exchange reactions using φX174 ssDNA and dsDNA substrates were carried out as described (32), with minor modifications. All reaction steps were carried out at 37°C. The final volume of each reaction was 50 μl. All concentrations given are final concentrations. RAD51 WT, D149N, R150Q or G151D protein (7.5 μM) was preincubated with 30 μM φX174 ssDNA for 5 min in buffer containing 40 mM Tris–HCl (pH 7.8), 1 mM MgCl_2_, 100 mM KCl, 1 mM DTT, 2 mM ATP, 8 mM creatine phosphate and 28 μg/ml creatine phosphokinase. Human RPA protein (2 μM) was added followed by a 5 min incubation, then 100 mM ammonium sulfate was added followed by a 1 min incubation. Reactions were initiated by the simultaneous addition of 30 μM (15 μM basepairs) linear φX174 dsDNA and 4 mM spermidine. At the indicated times, 6.5 μl aliquots were removed and brought to a final concentration of 0.8% SDS and 0.8 mg/ml Proteinase K, then incubated at 37°C for 15 min. Following addition of 10% glycerol and a trace of bromophenol blue, the samples were loaded onto a 0.9% agarose gel in Tris-acetate-EDTA (TAE) buffer and electrophoresed for 16 h at 25 V. Gels were stained with 1 μg/ml Sybr Gold and digitally photographed to record a negative image. Results were quantified by measuring the intensity of the joint molecule and nicked circle product bands relative to total DNA by using a Bio-Rad Personal Molecular Imager-FX with Quantity One (Bio-Rad) software (Vermont Cancer Center DNA Analysis Facility). Some reactions were modified to contain 250 mM KCl, and/or to contain a 1:1 mixture of RAD51 WT and G151D variant proteins (7.5 μM total concentration).

### Electron microscopy

Transmission electron microscopy was carried out in the University of Vermont College of Medicine Microscopy Imaging Facility. Protein–ssDNA complexes were assembled by incubating M13mp18 ssDNA (3 μM) and RAD51 WT or variant proteins (4 μM) in 25 mM triethanolamine-HCl buffer (pH 7.2) containing 2.5 mM magnesium acetate, 1 mM DTT and 1 mM ATP at 37°C for 15 min. Samples were applied to carbon-coated grids previously treated by glow discharge and stained with 2% uranyl acetate. Filaments formed by RAD51 WT, D149N, R150Q and G151D were visualized in a JEOL JEM-1210 transmission electron microscope at 60 kV and a magnification of 100 000X.

### Electrophoretic mobility shift assays

The properties of RAD51 and variant complexes with circular φX174 ssDNA and linear φX174 dsDNA were examined by electrophoretic mobility shift. Protein–ssDNA complexes were assembled in buffer containing 25 mM HEPES (pH 7.5), 1 mM MgCl_2_, 30 mM NaCl, 1 mM dithiothreitol, 0.4 mM 2-mercaptoethanol, 0.03 mM EDTA, 0.1 mg/ml BSA, 2% glycerol and 1 mM ATP. For protein–dsDNA complex assembly, the NaCl concentration was increased to 45 mM. RAD51 WT, D149N, R150Q and/or G151D proteins (0–8 μM depending on experiment) were incubated with φX174 ssDNA or dsDNA (20 μM) for 15 min at 37°C. Samples were loaded onto a 0.8% agarose gel in Tris–acetate–EDTA buffer, then electrophoresed for 16 h at 25 V. The gel was stained with 1 μg/ml Sybr Gold, trans-illuminated with UV light and digitally photographed.

### Sequence and structural analyses

Protein sequences were aligned using BLAST (http://blast.ncbi.nlm.nih.gov/Blast.cgi). Residues 121–167 of *Homo sapiens* RAD51 protein (HsRAD51; CAG38796.1) were used to query orthologs from *Mus musculus* (MmRad51; BAA02718.1), *Danio rerio* (DrRad51; AAH62849.1), *Drosophila melanogaster* (DmRad51; BAA04580.1), *Schizosaccharomyces pombe* (SpRhp51; CAB90141.1), *Saccharomyces cervisiae* (ScRad51; CAA45563.1) and *Pyrococcus furiosus* (PfRadA; AFN04713.1) and to *H. sapiens* DMC1 protein (HsDMC1; CAG30372.1). All structural figures of human RAD51 and yeast Rad51 proteins and site-directed mutants were prepared using PyMOL (The PyMOL Molecular Graphics System, Version 1.5.0.4 Schrödinger, LLC.). Structures of monomeric human RAD51 core domain and core domain-BRC4 complex were derived from PDB ID: 1N0W (33). Structures of filamentous yeast Rad51 protein were derived from PDB ID: 3LDA (34).

## RESULTS

### Discovery of human RAD51 somatic variants D149N and G151D

Exon 6 of the *RAD51* gene was targeted for sequence analysis because of the previous report of the R150Q variant, which occurs in this exon (30). Exon 6 was amplified from the genomic DNA of 32 freshly frozen breast tumor samples, each from different patients. Two of the thirty-two samples were found to have a mutation in exon 6. One of the mutations, G151D, resulted in altering Gly at position 151 to Asp (ggt to gat). It was found in an African American premenopausal woman who had local radiation failure. The type of breast cancer was unknown. The second mutation, D149N, resulted in altering Asp at position 149 to Asn (gac to aac). It was found in a premenopausal Caucasian woman with triple negative breast cancer. G151D was heterozygous; D149N was homozygous. Both mutations occur at positions adjacent to a previously reported R150Q mutation that was found in patients with bilateral breast cancer (12,30). Therefore mutations in three consecutive residues in a single motif of RAD51 protein are associated with breast cancer.

### Structural context of tumor-associated mutations in RAD51 protein

**Figure 1.**
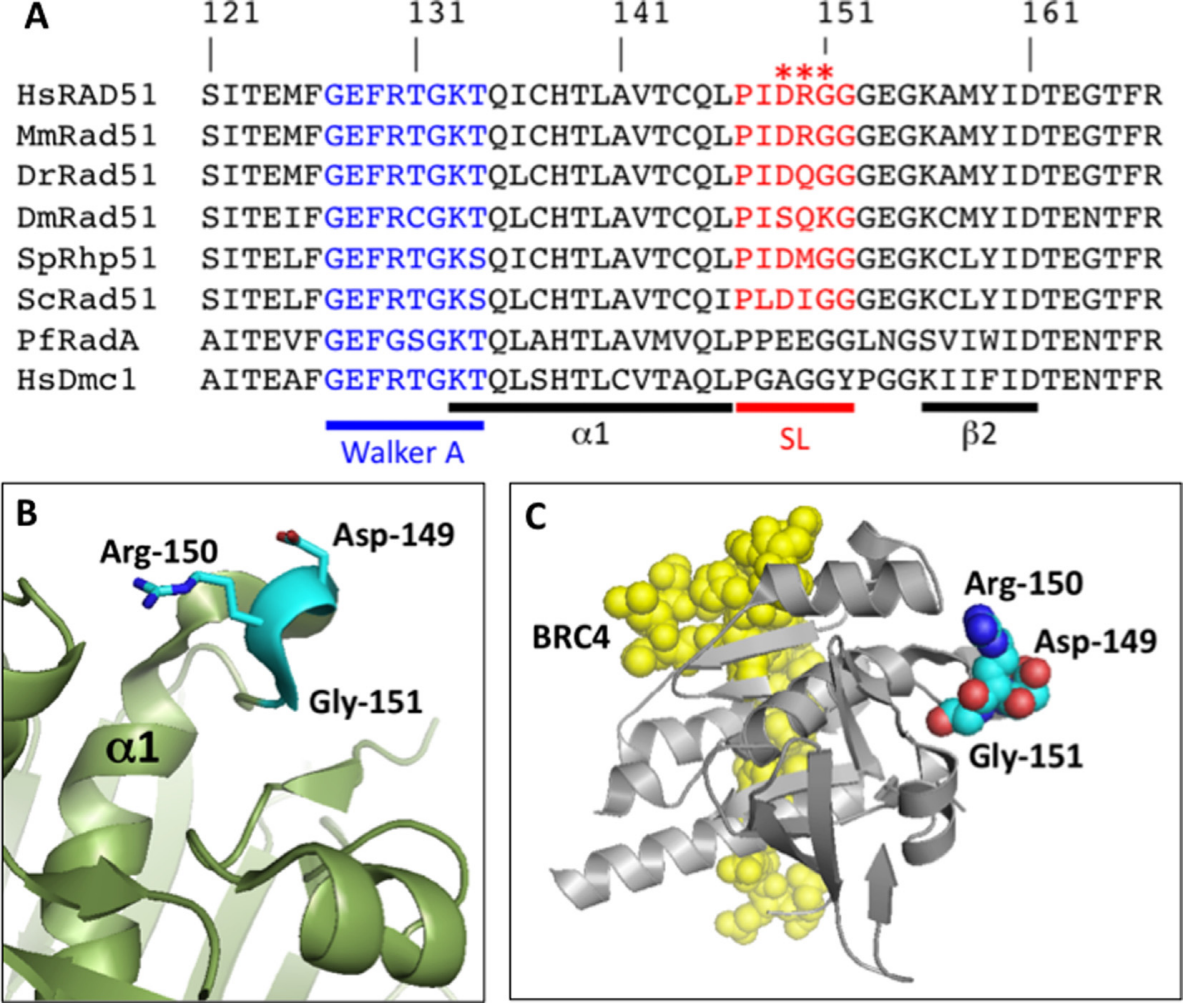
Sequence alignment and structure of the Schellman loop region of human RAD51 protein. (**A**) BLAST alignments of the Schellman loop region of *Homo sapiens* RAD51 protein (HsRAD51) to orthologs from *Mus musculus* (MmRad51), *Danio rerio* (DrRad51), *Drosophila melanogaster* (DmRad51), *Schizosaccharomyces pombe* (SpRhp51), *Saccharomyces cervisiae* (ScRad51) and *Pyrococcus furiosus* (PfRadA), and to *Homo sapiens* DMC1 protein (HsDMC1). The position of the conserved Schellman loop (SL) motif is indicated in red. Residues 149–151 in human RAD51, which are mutated in tumor-associated variants D149N, R150Q and G151D, are marked by red asterisks. The position of the Walker A motif is indicated in blue. Sequences corresponding to α-helix 1 (α1) and β-strand 2 (β2) are indicated by the black bars. (**B**) Structure of the Schellman loop region of human RAD51. Residues Asp-149, Arg-150 and Gly-151, which are mutated in tumor-derived variants, are highlighted in cyan. (**C**) Structure of the human RAD51–BRC4 complex, showing the relative positions of the BRC4 peptide (yellow, space-filling) and the RAD51 core domain (silver, cartoon) with Asp-149, Arg-150 and Gly-151 residues highlighted (colored by atom, space-filling). Structures in (B) and (C) were rendered from PDB ID: 1N0W.

**Figure 2.**
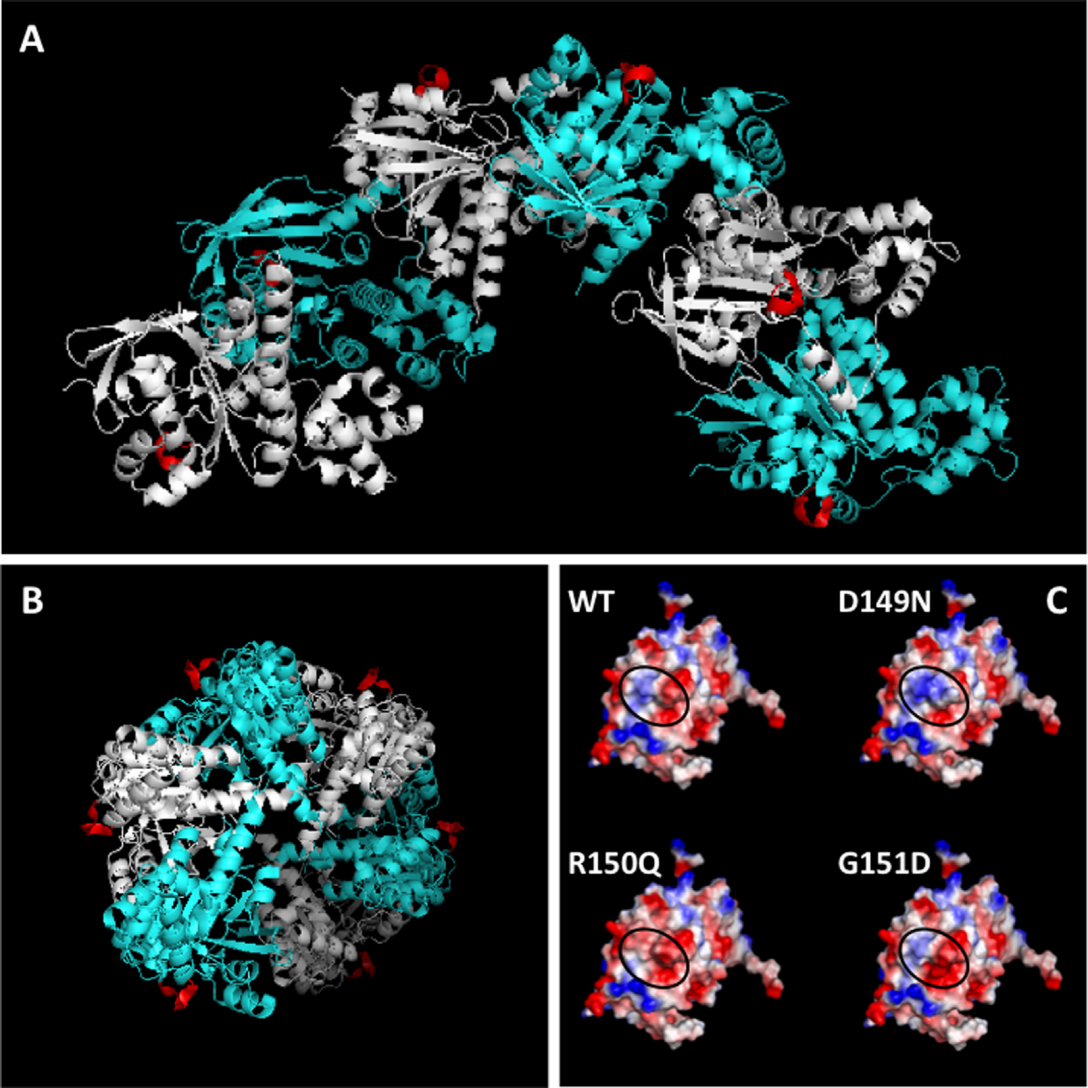
Location of the Schellman loop motif on the yeast Rad51 filament. (**A**) Structure of six contiguous subunits (one helical turn) of the *Saccharomyces cerevisiae* Rad51 filament (PDB ID: 3LDA), viewed perpendicular to the filament screw axis, with alternating subunits shown in cyan and silver. The Schellman loop motif on each subunit is highlighted in red. (**B**) End view, looking down the filament screw axis, which is perpendicular to the plane of the page. Color scheme as in (A), above. (**C**) Structure of the WT RAD51 core domain from PDB ID: 1N0W was altered to D149N, R150Q or G151D, and the resulting surface potentials were calculated. Blue denotes electropositive character, red denotes electronegative character and white denotes neutral character. The WT and variant proteins are shown in the same orientation. In each case, the black oval indicates the region of the protein surface corresponding to the Schellman loop.

The three mutations D149N, R150Q and G151D occur in the highly conserved RecA homology domain of RAD51. This domain contains the catalytic core of RAD51 protein including Walker A and B consensus sequences for ATP binding and hydrolysis, single- and double-stranded DNA binding surfaces (loops L1 and L2), polymerization motifs and recognition sites for other recombination factors and regulators including tumor suppressor proteins BRCA2, PALB2 and p53 (19,35–37). The three mutations occur within the sequence PIDRGG from positions 147 to 152, a motif that is highly conserved in Rad51 proteins from yeast to humans (Figure 1A). This sequence forms a Schellman loop (38) that connects the C-terminus of α-helix 1 to the N-terminus of β-strand 2 in the RecA homology domain (Figure 1B). The Schellman loop is seen in X-ray crystallographic structures of both monomeric human RAD51 core domain (Figure 1B and C) (33), and filamentous yeast Rad51 (Figure 2A-B) (34), and it forms a prominent surface feature of both the monomer and filament. The average B-factor for the Schellman loop region is 13.47, which is lower than the overall value (21.10) for the RAD51 monomer (Supplementary Figure S1). Therefore, the motif in which the three tumor-associated mutations occur has rather low freedom of movement, indicating that its position in RAD51 structure is relatively conservative.

The Schellman loop motif is distant from the binding site for the BRC4 peptide of BRCA2, as shown in the crystal structure of the RAD51 core domain-BRC4 fusion protein (Figure 1C). In the structure of the yeast Rad51 filament, the Schellman loop is found on the outer filament surface (Figure 2A and B). It is distant from the filament interface and thus from the active site for ATP binding and hydrolysis, which spans the interface and involves residues from adjacent subunits (34,39). It is also distant from DNA binding loops L1 and L2, which line the inner surface of the helical filament (40).

All three of the tumor-associated mutations alter the local surface electrostatic properties of RAD51. The D149N, R150Q and G151D mutations change the net charge on RAD51 by +1, −1 and −1 units, respectively. The D149N mutation significantly increases the electropositive character of the surface created by the Schellman loop (Figure 2C). In contrast, both R150Q and G151D mutations significantly increase the electronegative character of the same region of the protein surface (Figure 2C). Each of these changes, when propagated throughout a RAD51 filament, might be expected to alter interactions of the filament with solvent, ions or protein partners.

### Folding and stability of RAD51 variant proteins

Recombinant RAD51 variant proteins D149N, R150Q and G151D were expressed and purified, and their biochemical properties were compared to those of WT RAD51 protein. The folding and stability of RAD51 variants were assessed by circular dichroism spectroscopy and partial proteolysis. CD spectra of RAD51 WT, D149N and R150Q obtained in the presence and absence of ATP were essentially identical (Supplementary Figure S2A and B), therefore these variants share the same overall fold as WT. The G151D CD spectra were also similar in shape and amplitude to that of WT, but there were subtle differences. In the absence of ATP, the G151D spectrum was red-shifted by approximately 2 nm in the 190–222 nm region (Supplementary Figure S2A). This effect was also seen in the presence of 1 mM ATP, and a minimum at ∼208 nm became slightly more pronounced (Supplementary Figure S2B). One possible interpretation of this result is that the G151D mutation extends the helical turn portion of the Schellman loop, and that this adds helical character to the CD spectra. Such an effect is not reflected in the numerical analysis of the CD spectra, however, which revealed no significant changes in α-helical composition between WT and all three mutant forms of RAD51, independent of ATP (Supplementary Table S1). It seems probable that changes in the secondary structure of the Schellman loop trigger other minor changes in secondary structure without affecting the overall protein fold.

Protein thermal stability was monitored by recording the change in molar ellipticity at 222 nm as a function of temperature from 10–80°C (Supplementary Figure S2C). No significant changes in thermal stability were observed between the three variants and WT. Similar results were obtained using urea rather than heat as the denaturant (data not shown). Limited trypsinolysis of RAD51 WT and variants produced nearly identical fragment patterns (Supplementary Figure S3). In all cases, addition of a nucleotide ligand (ATP, ADP or ATPγS) caused the appearance of additional bands at ∼15 and ∼31 kDa. Taken together, the CD and partial proteolysis data indicate that the three tumor-associated variants are folded and stable.

### Kinetics of RAD51 variant-catalyzed ATP hydrolysis

**Figure 3.**
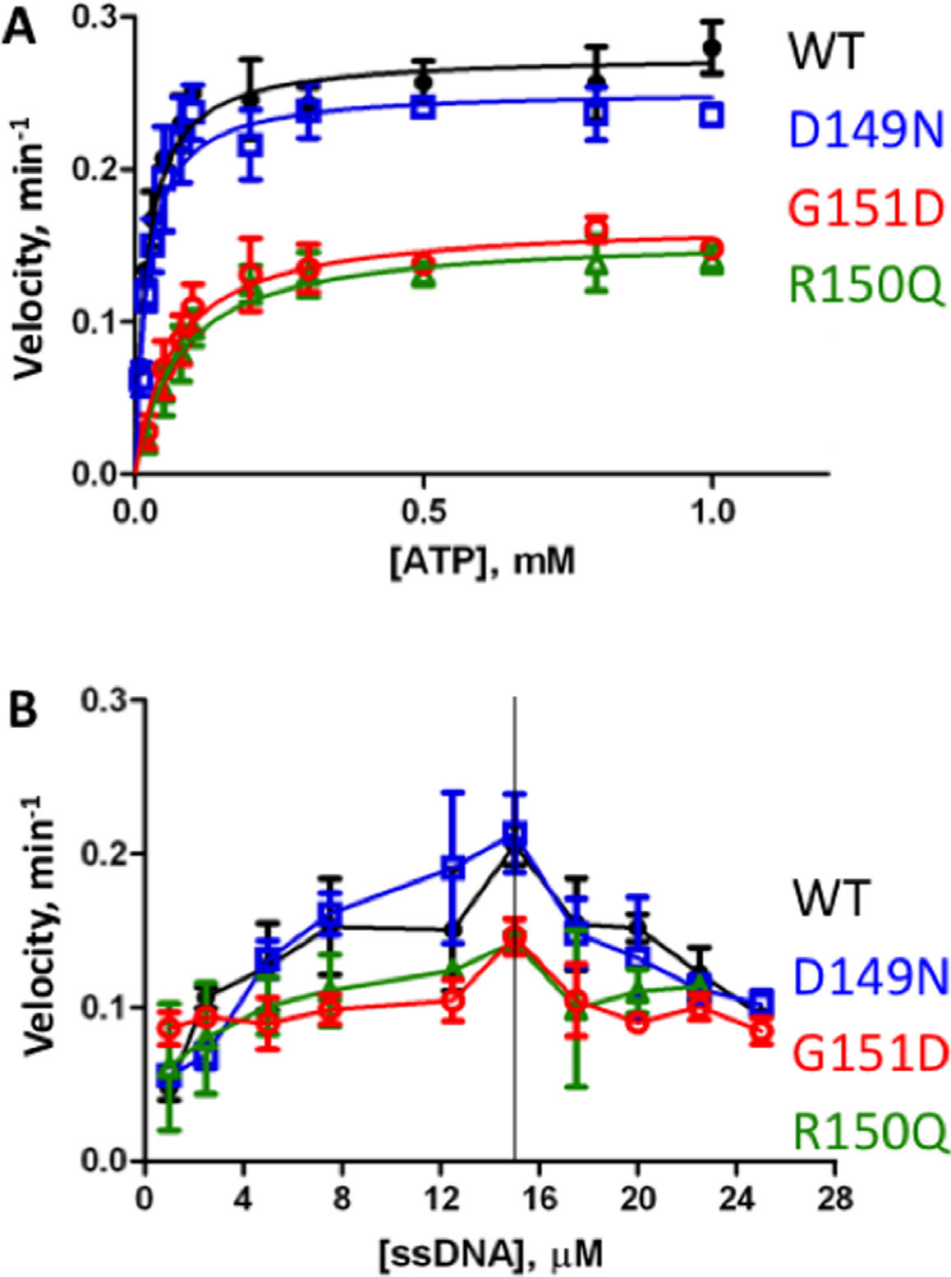
Steady-state kinetics of ATP hydrolysis by variant and WT RAD51 proteins in the presence of ssDNA. Steady-state velocities were measured as described in ‘Materials and Methods’ section. Data points and error bars represent averages and standard deviations from three experiments. Data for RAD51 WT, D149N, R150Q and G151D are shown in black, blue, green and red symbols and lines, respectively. (**A**) Reaction velocity as a function of ATP concentration. ssDNA (6 μM) and protein (2 μM) were present at a ratio of 3 nucleotide residues per protomer, which is equivalent to the binding site size of RAD51 on ssDNA. (**B**) Reaction velocity as a function of ssDNA concentration. A constant concentration of 2 μM protein and a constant, saturating concentration of 1 mM ATP was maintained, while the concentration of ssDNA was varied.

RAD51 protein is fundamentally an ATPase that is stimulated by ssDNA. The cycle of ATP binding and hydrolysis is linked to the assembly and disassembly of RAD51 nucleoprotein filaments on DNA, and to DNA strand exchange activity (19). We determined the ATPase properties of tumor-derived RAD51 variants by performing steady-state kinetics studies with either ATP or ssDNA concentration as the variable (Figure 3).

With ATP as the variable substrate and at a constant, stoichiometric concentration of ssDNA with respect to protein (3 nucleotide residues per protomer), we noted large differences in the ATPase kinetics of R150Q and G151D compared to WT, while the ATPase kinetics of D149N were similar to WT (Figure 3A). At saturating ATP concentrations, R150Q and G151D exhibited nearly identical catalytic rates, both of which were substantially lower than WT (Figure 3A). In contrast, the activity of D149N was similar to WT at saturating ATP. Neither WT nor any of the variants showed any sign of ATP-induced cooperativity. Therefore a standard Michealis–Menten model was used to fit the data. Values for the kinetic parameters are listed in Table 2. The data indicate that the R150Q and G151D mutations decrease the turnover rate (*k*_cat_) by nearly 2-fold compared to WT, while the *K*_m_ for ATP increases by 3- to 4-fold. The combination of these effects leads to a catalytic efficiency (*k*_cat_/*K*_m_) for ATP hydrolysis that is reduced by 6- to 7-fold for R150Q and G151D compared to WT (Table 2). In contrast, the *K*_m_ of D149N for ATP is identical to that of WT, and its turnover and catalytic efficiency parameters are within 90% of WT (Table 2).

**Table 2.** Steady-state kinetic parameters of RAD51-catalyzed ATP hydrolysis in the presence of ssDNA

	WT	D149N	R150Q	G151D
*k*_cat_ (min^−1^)	0.28 ± 0.01	0.25 ± 0.01	0.16 ± 0.01	0.17 ± 0.01
*K*_m_ (μM ATP)	20 ± 3	20 ± 3	76 ± 12	67 ± 9
*k*_cat_/*K*_m_ (s^−1 ×^ M^−1^)	230	210	34	41

All data were derived from computer fits of steady-state ATPase curves as shown in Figure 3A and as described in ‘Materials and Methods’ section. Each value represents the average (± standard deviation) from three different experiments.

We considered the possibility that the ATPase defects seen in R150Q and G151D could be caused by inefficient binding to ssDNA. Therefore in the experiments in Figure 3B the ATP concentration was held at a constant, saturating level and the concentration of ssDNA was varied. The ATPase activity of WT RAD51 exhibits a ssDNA concentration optimum, above which the reaction velocity declines with increasing ssDNA concentration (Figure 3B). Furthermore, all three of the RAD51 variants have their highest ATPase activities at the same ssDNA concentration as WT (Figure 3B). This rules out the possibility that the observed differences in kinetic parameters between R150Q, G151D and WT (Figure 3A, Table 2) are caused by inefficient binding of the variants to ssDNA, since additional ssDNA does not compensate for the defect. Note that unlike WT and the other two variants, the ATPase activity of G151D appears to be independent of ssDNA at low concentrations of ssDNA (Figure 3B). This suggests that G151D maintains a higher-than-normal constitutive ATPase activity that is relatively insensitive to further activation by ssDNA.

### DNA strand exchange properties of RAD51 variants

**Figure 4.**
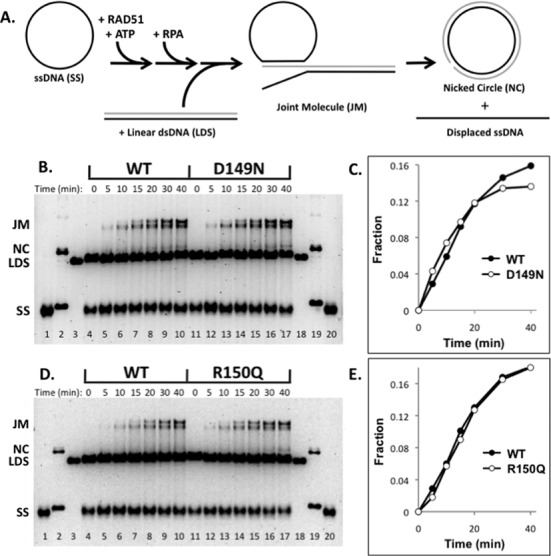
DNA strand exchange activities of D149N and R150Q variants compared to WT RAD51. DNA strand exchange assays were carried out as described in ‘Materials and Methods’ section, using normal salt conditions (100 mM KCl). (**A**) Reaction schematic. Homologous φX174 circular ssDNA (SS) and linear dsDNA (LDS) substrates are converted by RAD51 protein into heteroduplex products including joint molecules (JM) and nicked circles (NC), which, following deproteination, are resolved from substrates by electrophoresis on agarose gels. The staging of the reaction involves the preincubation of RAD51 with SS in the presence of ATP, followed by the addition of RPA, followed in turn by the addition of LDS to start the reaction. See ‘Materials and Methods’ section for details. (**B**) Comparison of D149N and WT activities. *Lanes 1–3* and *18–20* contain markers for SS, NC and LDS, respectively. *Lanes 4–10* show the timecourse (0–40 min) of DNA strand exchange catalyzed by WT following reaction initiation by the addition of LDS. *Lanes 11–17* show the timecourse (0–40 min) of DNA strand exchange catalyzed by D149N following reaction initiation by the addition of LDS. (**C**) Quantification of strand exchange products from reactions with D149N and WT, shown in panel (B). (**D**) Comparison of R150Q and WT activities. *Lanes 1–3* and *18–20* contain markers for SS, NC and LDS, respectively. *Lanes 4–10* show the timecourse (0–40 min) of DNA strand exchange catalyzed by WT following reaction initiation by the addition of LDS. *Lanes 11–17* show the timecourse (0–40 min) of DNA strand exchange catalyzed by R150Q following reaction initiation by the addition of LDS. (**E**) Quantification of strand exchange products from reactions with R150Q and WT, shown in panel (D).

**Figure 5.**
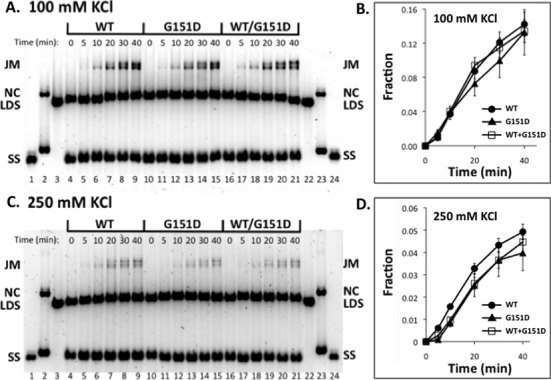
Effects of salt and mixing with WT on the DNA strand exchange activity of RAD51 G151D variant. DNA strand exchange assays were carried out as described in ‘Materials and Methods’ section. Reactions were staged as described in Figure 4A. (**A**) Activities of WT, G151D and WT/G151D mixture in 100 mM KCl. *Lanes 1–3* and *22–24* contain markers for SS, NC and LDS, respectively. *Lanes 4–9* show the timecourse (0–40 min) of DNA strand exchange catalyzed by WT following reaction initiation by the addition of LDS. *Lanes 10–15* show the timecourse (0–40 min) of DNA strand exchange catalyzed by G151D following reaction initiation by the addition of LDS. *Lanes 16–21* show the timecourse (0–40 min) of DNA strand exchange catalyzed by a 1:1 mixture of WT and G151D, following reaction initiation by the addition of LDS. The total concentration of WT + G151D variant was constant in all reactions. (**B**) Quantified results from reactions at 100 mM KCl. Vertical axis denotes the fraction of total DNA appearing as joint molecule and nicked circle products. Standard error from two experiments is shown. (**C**) Identical to panel (A), except that reactions contained 250 mM KCl. (**D**) Quantified results from reactions at 250 mM KCl. Standard error from two experiments is shown.

The D149N, R150Q and G151D variants are all proficient in DNA strand exchange (Figures 4 and 5). Strand exchange activities were tested using full-length (5.4 kb/kbp) ssDNA and dsDNA substrates derived from bacteriophage φX174. Reactions were carried out in the presence of human RPA protein and 2 mM ATP. The ability of RAD51 variants to carry out strand exchange under these conditions indicates that they retain sufficient ssDNA binding affinity to compete with RPA protein for binding sites on ssDNA. The kinetics of joint molecule formation by the D149N (Figure 4B and C) and R150Q (Figure 4D and E) variants during strand exchange was in all cases similar to WT RAD51. Similar reactions carried out at an elevated salt concentration (250 mM KCl) or at reduced ATP concentration (0.2 mM) revealed no significant differences in strand exchange activity between D149N, R150Q and WT (data not shown).

Under standard reaction conditions (100 mM KCl) the rate of DNA strand exchange catalyzed by G151D alone, or by a 1:1 mixture of G151D and WT, did not differ significantly from the rate observed with WT alone (Figure 5A and B). In 250 mM KCl, the rate and extent of strand exchange catalyzed by G151D were slightly decreased compared to WT (Figure 5C-D), suggesting a slight increase in the salt-sensitivity of this variant compared to WT. Also in 250 mM KCl, a 1:1 mixture of G151D and WT (while maintaining an equivalent total RAD51 concentration) exhibited strand exchange activity that was similar to that of G151D alone (Figure 5C and D). This finding suggests that G151D could affect recombination activity in a cell that is heterozygous for the variant.

### Properties of RAD51 variant nucleoprotein complexes

**Figure 6.**
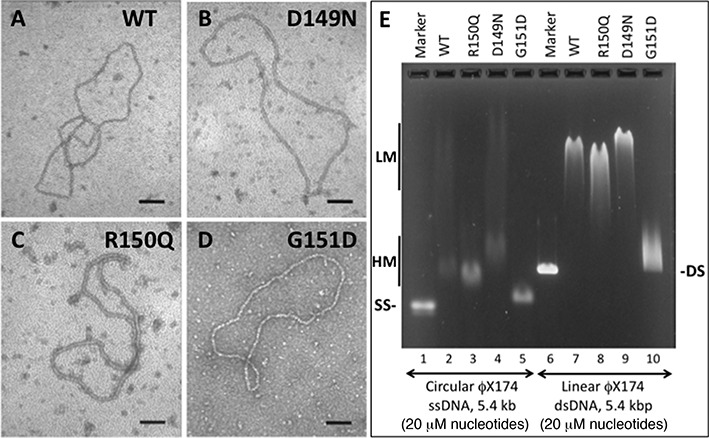
Properties of hRAD51 variant nucleoprotein filaments. (**A**–**D**) Electron micrographs of representative nucleoprotein filaments formed by variant and WT RAD51 proteins on φX174 circular ssDNA in the presence of ATP. All images are 100 000X magnification. The black scale bar in each panel represents a distance of 100 nm. (**E**) Differential electrophoretic mobilities of complexes formed by variant and WT RAD51 proteins on φX174 circular ssDNA and linear dsDNA in the presence of ATP. *Lanes 1* and *6* contain markers for naked ssDNA and dsDNA, respectively. *Lanes 2–5* contain WT, R150Q, D149N and G151D complexes with ssDNA, respectively. *Lanes 7–10* contain WT, R150Q, D149N and G151D complexes with dsDNA, respectively. ‘LM’ and ‘HM’ denote low- and high-mobility protein–ssDNA complexes, respectively.

In the presence of ATP, variant proteins D149N, R150Q and G151D all form filaments on single-stranded DNA that resemble those formed by WT RAD51, as shown by electron microscopy (Figure 6A and D). The micrographs show that all four versions of RAD51 are capable of saturating M13mp18 ssDNA circles under the low-salt conditions used to form and fix complexes for EM imaging. The filaments all exhibit the zigzag pattern that is characteristic of helical RAD51–DNA filaments. Qualitatively, we noted that filaments formed by WT and D149N tended to appear segmented and stiff (Figure 6A and B), while those formed by G151D (Figure 6D) and to an intermediate extent R150Q (Figure 6C) tended to appear as smooth coils. This raises the possibility that the mutations may differentially affect filament stiffness.

The D149N, R150Q and G151D mutations have different and dramatic effects on the electrophoretic mobility of nucleoprotein complexes formed by RAD51 in the presence of ATP (Figure 6E). At a protein concentration that is approximately stoichiometric with respect to DNA concentration (1 protomer per 3 nucleotide residues), WT RAD51–ssDNA complexes migrate in two distinct fractions with high and low electrophoretic mobility (HM and LM, respectively) (Figure 6E, lane 2). The same pattern is seen with D149N–ssDNA, but the mobilities of both HM and LM fractions are decreased compared to WT (Figure 6E, lane 4). With R150Q–ssDNA, nearly all of the complexes migrate in the HM fraction, which has slightly higher mobility than the WT equivalent (Figure 6E, lane 3). Similar behavior was noted for R150Q–DNA complexes in a previous study (30). G151D–ssDNA complexes appear to migrate exclusively in the HM fraction, which has much higher electrophoretic mobility than the WT equivalent (Figure 6E, lane 5).

Binding of RAD51 WT and variants to duplex DNA in the presence of ATP results in single smeared bands rather than the LM and HM species observed with ssDNA (Figure 6E). However, differences between variants and WT follow the same pattern seen with ssDNA: D149N–dsDNA migrates slower than the WT complex (Figure 6E, lane 9); R150Q–dsDNA migrates faster (Figure 6E, lane 8) and G151D–dsDNA migrates much faster (Figure 6E, lane 10).

Differences in the electrophoretic mobilities of RAD51 variant–DNA complexes could be explained by differences in filament electrostatic properties, stiffness, stability or a combination of these factors. Formation of WT, D149N and R150Q complexes on ssDNA and dsDNA show similar dependencies on protein concentration in the presence of ATP, ADP or no nucleotide (Supplementary Figure S4). The same may be true for G151D–ssDNA, although the trend is harder to see because of the small difference in electrophoretic mobility between G151D–ssDNA and naked ssDNA (Supplementary Figure S4). The magnitude of the ssDNA mobility shift increases steadily with increasing G151D concentration in the presence of ADP or ATP, and the complexes form tight bands, not smears (Supplementary Figure S4B and C). G151D–dsDNA complex formation appears to require ATP (Supplementary Figure S4D–F), and mobility shift is only observed at higher G151D concentrations (Supplementary Figure S4F). Collectively, the electrophoretic mobility shift data indicate that, with the possible exception of G151D–dsDNA interactions, differences in the electrophoretic properties of RAD51 variant–DNA complexes are not driven by large differences in DNA binding affinity. This general conclusion is supported by other observations including: (i) variants including G151D can saturate long ssDNA molecules by forming continuous filaments (Figure 6B–D), (ii) maximum ATPase rates of variants and WT occur at the same ssDNA/protein ratio (Figure 3B) and (iii) Variant strand exchange activities approach those of WT in the presence of RPA (Figures 4 and 5), indicating that all variants compete effectively with RPA for binding sites on ssDNA.

### Variants can form mixed filaments with WT RAD51

**Figure 7.**
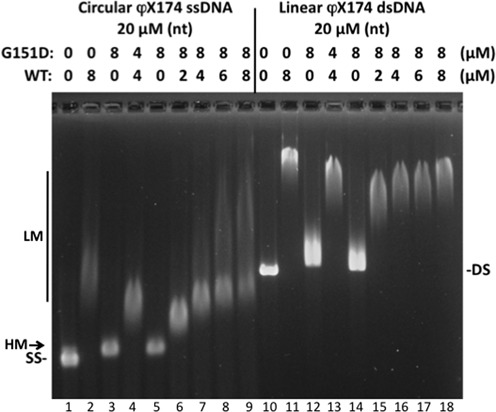
G151D and WT RAD51 form mixed complexes on ssDNA and dsDNA. Electrophoretic mobility shift assays were performed as described in Figure 6E, except that the concentrations and ratios of RAD51 WT and G151D proteins were varied as indicated. *Lane 1*, ssDNA marker. *Lane 2*, ssDNA + 8 μM WT. *Lane 3*, ssDNA + 8 μM G151D. *Lane 4*, ssDNA + 4 μM WT + 4 μM G151D. *Lanes 5–9*, ssDNA + 8 μM G151D + 0, 2, 4, 6 and 8 μM WT, respectively. *Lane 10*, dsDNA marker. *Lane 11*, dsDNA + 8 μM WT. *Lane 12*, dsDNA + 8 μM G151D. *Lane 13*, dsDNA + 4 μM WT + 4 μM G151D. *Lanes 14–18*, dsDNA + 8 μM G151D + 0, 2, 4, 6 and 8 μM WT, respectively. ‘LM’ and ‘HM’ denote low- and high-mobility protein–ssDNA complexes, respectively.

Variants D149N, R150Q and G151D all interact with WT RAD51 protein as shown in yeast two-hybrid experiments (Supplementary Figure S5). This suggests that variants could form mixed filaments with WT RAD51 on DNA. We tested this possibility using the G151D variant, which shows the greatest difference from WT in EMSA assays (Figure 7). In the presence of ATP, complexes formed with 20 μM ssDNA and a 4 + 4 μM mixture of G151D and WT (Figure 7, lane 4) have an electrophoretic mobility that is intermediate between the complex formed with 20 μM ssDNA plus 8 μM G151D alone (Figure 7, lane 3) and the complex formed with 20 μM ssDNA plus 8 μM WT alone (Figure 7, lane 2). Note that all of the ssDNA in the mixed experiment undergoes an intermediate mobility shift (Figure 7, lane 4). In the case where ssDNA is saturated with G151D and increasing amounts of WT are added (Figure 7, lanes, 5–9), we observe that WT can partially replace G151D to generate complexes with intermediate electrophoretic mobility. Note again that, at the lower concentrations of WT RAD51 added, all or most of the ssDNA undergoes an intermediate mobility shift even though the total protein concentration is above stochiometric for binding sites on ssDNA (Figure 7, lanes 6–7). The same behavior is observed with duplex DNA, where mixtures of G151D and WT RAD51 form complexes with intermediate electrophoretic mobility (Figure 7, lanes 13 and 15–18). The data demonstrate that the binding of G151D does not exclude WT RAD51 binding to the same DNA molecule or *vice versa*. Combined with the yeast two-hybrid data (Supplementary Figure S5), and with strand exchange data for the G151D/WT mixture in high salt (Figure 5D), these results strongly argue that RAD51 variants can incorporate into filaments along with WT to form nucleoprotein filaments with altered properties. This finding may be significant for understanding the behavior of RAD51 variants in the heterozygous state in cancer cells, as occurs in the case of G151D.

## DISCUSSION

### Significance of mutations in RAD51 versus its regulators

Tumor-associated variants of human RAD51 protein, D149N, R150Q and G151D, are proficient in DNA strand exchange activity but have altered physical and biochemical properties. Given the location of these mutations on the surface of RAD51 nucleoprotein filaments, they are candidates to affect the regulation of RAD51, or the coordination of its activities with other DNA recombination/repair proteins. In normal human cells, recombination events promoted by RAD51 protein are closely regulated to prevent genotoxic effects caused by inappropriate crossovers, break-induced replication and loss of heterozygosity (41). Cancer cells frequently exhibit dysfunctional regulation of HR, which may be linked to somatic or germ-line mutations in proteins that regulate RAD51, such as BRCA2, PALB2, BLM, p53 and others (19,35–37). These factors interact with RAD51 to regulate the assembly, activity and turnover of RAD51–DNA nucleoprotein filaments. It is reasonable to conclude that mutations in RAD51 that affect its interactions with regulatory molecules may be just as deleterious as mutations in the regulators themselves. In many cases the target for regulation is the RAD51–DNA *filament*, therefore signals from regulatory molecules must be transmitted throughout the filament. For this reason, mutations that affect endogenous signaling within the filament may be just as deleterious as mutations that affect signaling by exogenous regulators of RAD51. It is intriguing that the three variants described in this work alter properties of RAD51 filaments that could affect either endogenous or exogenous signaling, or both.

### Potential effects of RAD51 mutations on exogenous signaling

The remarkable occurrence of D149N, R150Q and G151D mutations in the same Schellman loop motif means that each mutation alters what is a prominent feature of the surface of the RAD51 filament (Figures 1 and 2). Each mutation affects the local electrostatic properties of the filament surface, with D149N more electropositive, and R150Q and G151D more electronegative than WT (Figure 2C). The mutations occur on the outer filament surface, where they could disrupt interactions with other recombination proteins and regulators. One candidate is tumor suppressor protein p53, which was reported to interact directly with RAD51 and to negatively regulate its DNA pairing activity (42). RAD51 peptides containing residues 175–190 were shown to bind to p53 (37). In the X-ray crystallographic structure of the RAD51 core domain–BRC4 complex (33), the side chain of Arg-150 interacts with this site via a hydrogen bond to the backbone of residue Tyr-178 (Supplementary Figure S6). This raises the possibility that mutations affecting Arg-150 or its neighbors in the Schellman loop motif could affect regulatory interactions between RAD51 and p53. The Schellman loop motif is not directly involved in binding to BRCA2 via its BRC repeats (Figure 1C, Supplementary Figure S6), or to PALB2 which is reported to interact with residues 184–257 of RAD51 (36) (Supplementary Figure S6). This does not rule out an indirect effect of mutations in the Schellman loop on interactions with these tumor suppressor proteins, however.

### Tumor-associated mutations alter physical properties of RAD51 nucleoprotein filaments

Changes in the electrostatic surface potentials of the D149N, R150Q and G151D variants correlate with changes in the physical properties of their nucleoprotein filaments on both ssDNA and dsDNA. Complexes containing the more electropositive D149N variant exhibit reduced electrophoretic mobility, while those containing the more electronegative variants R150Q and G151D exhibit increased electrophoretic mobility compared to WT complexes (Figures 6E and 7). The latter two mutations may also relax filament stiffness (Figure 6C and D). Decreased stiffness and a more negative charge could explain the relatively rapid migration of G151D–DNA and R150Q–DNA complexes through agarose gels. In cells, the altered electrostatic and hydrodynamic properties of RAD51 variants could impact a variety of biophysical processes including the formation and resolution of RAD51 foci in response to DNA damage, the turnover of nucleoprotein filaments by helicases and other functions that involve the physical manipulation of RAD51 filaments.

### Mutations at distant sites alter endogenous signaling in RAD51 filaments

The DNA-stimulated ATPase cycle is the major pathway of endogenous signaling in RAD51 filaments. The nucleotide-bound status of the active site affects DNA binding and *vice versa*. Greater electronegative character in the Schellman loop region also correlates with changes in this activity, since both R150Q and G151D variants exhibit lower affinity for ATP, slower turnover and markedly reduced catalytic efficiency for ATP hydrolysis (Figure 3, Table 2). Our findings about R150Q appear to differ from those of a previous study, which found no difference in the ATPase activities of R150Q and WT RAD51 (30). However ATPase experiments in the earlier study were performed using an excess concentration of ssDNA, conditions in which WT and R150Q have very similar activities in our experiments (Figure 3B). Furthermore we observe that the DNA strand exchange activity of G151D appears to be slightly more salt-sensitive than WT (Figure 5). We conclude that mutations affecting the surface properties of RAD51–DNA filaments can affect reactions at distant active sites. This makes sense for a system that is allosteric in nature and dependent on filament formation for catalytic activity. Our findings suggest that computational approaches designed to identify deleterious variants based on non-conservative mutations in known functional motifs are likely to miss important mutations in RAD51 and perhaps many other allosteric proteins.

### Biochemical defects of the RAD51–G151D variant

Of the three variants examined, G151D stands out as the one most different from WT, both in terms of its biochemical properties and in its potential effects on the structure of the Schellman loop motif. Notably, G151D was detected as a somatic, heterozygous mutation in a patient with advanced breast cancer who had local radiation failure. While the G151D variant is folded and stable, features of its CD spectrum are consistent with subtle changes in secondary structure (Supplementary Figure S2A-B).

In addition to its lower ATPase catalytic efficiency (Figure 3A, Table 2), G151D appears to have a significant DNA-independent ATPase activity (Figure 3B). In WT RAD51, as in many recombinases of the RecA/Rad51 family, high-salt conditions induce a DNA-independent ATPase activity (43). The data suggest that this activity is constitutively active in G151D, even at the low salt concentrations used in our ATPase assays. We speculate that the altered surface electrostatics of the G151D variant allow it to assume an ATPase-active conformation that would normally be induced via interactions with ions. Our finding that the DNA strand exchange activity of G151D is slightly more salt-sensitive than WT (Figure 5C and D) supports the notion that this variant responds differently to ions, possibly due to its more electronegative surface character.

The extremely fast electrophoretic mobility of G151D–DNA relative to WT–DNA complexes (Figures 6E and 7) is consistent with the added negative charge of G151D, multiplied by thousands of protomers coating each DNA molecule and with a possible decrease in filament stiffness caused by the G151D mutation (Figure 6D). The affinity of G151D for ssDNA does not appear to differ greatly from that of WT, since the variant is capable of completely covering ssDNA in electron micrographs (Figure 6D), and since it catalyzes DNA strand exchange in the presence of RPA (Figure 5), with which it must compete for binding sites on ssDNA.

### Implications for heterozygous RAD51 variants

G151D interacts with WT RAD51 and forms mixed filaments with WT on both ssDNA and dsDNA (Supplementary Figure S5 and Figure 7). The hybrid filaments have intermediate electrophoretic mobility and DNA strand exchange properties (Figures 5C-D and 7). These findings indicate that in the heterozygous state in cells, as occurred in the breast tumor from which this variant was identified, G151D will co-localize with WT and form hybrid RAD51 foci, which may have altered HDR properties. The fact that D149N and R150Q variants also interact with WT RAD51 (Supplementary Figure S5) suggests that, if they were to occur in the heterozygous state *in vivo*, they too could form hybrid foci and potentially influence HDR processes.

### Conservation and divergence of the Schellman loop motif

The Schellman loop motif is conserved at the levels of sequence and structure among Rad51 proteins from yeast to humans (Figures 1A and 2). However charged residues Asp-149 and/or Arg-150 from human RAD51 are non-conservatively substituted in zebrafish, fruit fly and budding and fission yeast (Figure 1A). Therefore Rad51 filament surfaces in these organisms will have different electrostatic properties, which may lead to differences in filament dynamics or protein–protein interactions. In contrast, the loop region of human meiotic recombinase DMC1 consists of the simple sequence PGAGGY, which lacks charged/polar residues altogether (Figure 1A). This raises the possibility that differences in filament surface properties contribute to the functional divergence of DMC1 and RAD51 in meiotic recombination. The region of the Schellman loop appears to be important in archaeal Rad51 orthologs as well, since the structure of *Pyrococcus furiosus* RadA protein (PDB ID: 1PZN) (44) reveals a modified Schellman loop motif in which a mini-helix containing two Glu residues is separated from α-helix 1 by an extra Pro residue (Figure 1A).

In conclusion, the work presented here demonstrates that tumor-associated variants in human RAD51 have functional phenotypes including changes in the physical properties of RAD51–DNA filaments, and changes in the catalytic activities of these filaments. These changes have the potential to affect the efficacy of HDR, and therefore could contribute to the genome instability of cancer cells.

## ACCESSION NUMBERS

PDB IDs: 1N0W, 3LDA and 1PZN.

## SUPPLEMENTARY DATA

Supplementary Data are available at NAR Online.

SUPPLEMENTARY DATA
