# Supplementary material for: Tumor-associated mutations in a conserved structural motif alter physical and biochemical properties of human RAD51 recombinase
Source: Nucleic Acids Res. 2014 Dec 24;43(2):1098–111. doi: 10.1093/nar/gku1337 (PMC4333388; doi:10.1093/nar/gku1337)
Supplement: SUPPLEMENTARY DATA [file supp_43_2_1098__index.html]

Tumor-associated mutations in a conserved structural motif alter physical and biochemical properties of human RAD51 recombinase — SUPPLEMENTARY DATA 

# Tumor-associated mutations in a conserved structural motif alter physical and biochemical properties of human RAD51 recombinase

## SUPPLEMENTARY DATA

**Files in this Data Supplement:**

- SUPPLEMENTARY DATA
