# Supplementary material for: Tumor-associated mutations in a conserved structural motif alter physical and biochemical properties of human RAD51 recombinase
Source: Nucleic Acids Res. 2014 Dec 24;43(2):1098–111. doi: 10.1093/nar/gku1337 (PMC4333388; doi:10.1093/nar/gku1337)
Supplement: SUPPLEMENTARY DATA [file supp_gku1337_nar-02476-h-2014-File009.pdf]

Supplementary Data

Tumor-associated Mutations in a Conserved Structural Motif Alter Physical and Biochemical Properties of Human RAD51 Recombinase

Jianhong Chen, Milagros D. Morrical, Katherine A. Donigan, Joanne B. Weidhass, Joann B. Sweasy, April M. Averill, Jennifer A. Tomczak, and Scott W. Morrical

Supplementary Materials and Methods

Circular dichroism measurements. CD spectra were recorded at 37ºC on a Jasco 715 CD spectropolarimeter using a cuvette with a path length of 0.01 cm, a spectral bandwidth of 0.2 nm, and a time constant of 1 second. To obtain CD spectra, RAD51 samples (25-50 µM depending on variant) were pre-dialyzed into buffer containing 20 mM HEPES (pH 7.5), 1 mM $MgCl_2$, and 150 mM NaCl. 1 mM ATP was added to some samples prior to obtaining spectra. Each of the recorded spectra was the average of six scans to increase the signal-to-noise ratio. After normalization of signal for protein concentration and subtracting the blank signal for the cuvette and buffer, observed molar ellipticity (θ_d, in millidegrees) was converted to mean residue ellipticity ($deg*cm^2*dmol^{-1}$). The equation $\theta_{mr} = \theta_d*M/(c*l*n)$ was used, where M is the molecular weight, c is the molar residue concentration of protein, l is the path length of the cuvette in centimeters, n is the total number of amino acid residues. The software package CDPro (http://lamar.colostate.edu/~sreeram/CDPro/main.html) was used for determining the secondary structure fractions of proteins in the presence or absence of 1 mM ATP. As an indicator of the thermal stability of RAD51 variants, molar ellipticity at

222 nm was measured as a function of temperature. Protein concentration was 6 μM and the buffer was as described above, without added ATP.

Partial proteolysis assays. Protein conformational changes in response to nucleotide presence were assayed through partial proteolysis as reported with minor modification (45). In a total volume of 20 μl, 10 μg of RAD51 wild-type or variant protein was incubated in the buffer containing 20 mM Na-HEPES (pH 7.5), 150 mM NaCl, 10% glycerol, 1 mM dithiothreitol, 0.1 mM EDTA with 10 mM magnesium acetate and 1 mM ATP, ADP, or ATPγS. Reactions were incubated at 37 °C for 15 min in a Perkin-Elmer thermal cycler with heated lid. Then 6 μl aliquots were added to 6 μl 250 ng/μl trypsin. Reaction mixtures were incubated at 37 °C for another 60 min and reactions were stopped by the addition of 4 μl loading buffer containing 0.2 M Tris-HCl (pH 7.0), 8% SDS, 20% β-mercaptoethanol, 20% sucrose, 2 mg/ml bromophenol blue, and followed by 5 minutes boiling. Proteolytic products were resolved by 20% SDS-PAGE and visualized by staining with GelCode Blue Stain (Thermo Scientific). Gels were wrapped in clear plastic wrap and scanned directly with a Hewlett-Packard scanner.

Yeast two-hybrid assays. Wild-type human RAD51 coding sequence from plasmid pET-HsRad51 was PCR amplified and cloned into plasmid pDONR221 with BP Clonase II as described in the Gateway Cloning Technology Instruction Manual (Life Technologies). QuikChange site-directed mutagenesis was employed to generate the missense mutations D149N, R150Q, and G151D within the HsRad51/pDONR221 vector according to the manufacturer's instructions (Stratagene). Primers are shown in Table 1. Gateway Cloning (Life Technologies) was further utilized to shuttle each RAD51 insert into a bait (pDEST32) and a prey (pDEST22) vector. All plasmid inserts were sequenced to confirm they were

correct by the Vermont Cancer Center DNA Sequencing Facility. Yeast strain MaV203 was transformed with 1 μg of bait and 1 μg of prey vector by the lithium acetate and PEG method (46), and plated on synthetic complete-leu-trp plates. Transformants were selected and tested for β-galactosidase activity as described in the Proquest Two Hybrid System manual (Life Technologies). Briefly, colonies were streaked onto SC-leu-trp plates, grown overnight, and replica plated onto YPAD plates. After 24 hours at 30ºC, streaks were lifted onto nitrocellulose or nylon filters, immersed in liquid nitrogen, placed on Whatman paper soaked in X-gal solution, incubated overnight at 30º C and photographed. Blue streaks are indicative of interaction between proteins.

Supplementary References

45. Tombline, G., Heinen, C.D., Shim, K.S., and Fishel, R. (2002) Biochemical characterization of the human RAD51 protein. III. Modulation of DNA binding by adenosine nucleotides. *J. Biol. Chem.* **277**, 14434-14442.

46. Gietz, R.D. and Schiestl, R.H. (2007) High-efficiency yeast transformation using the LiAc/SS carrier DNA/PEG method. *Nat. Protoc.* **2**, 31-34.

	WT		D149N		R150Q		G151D	
	**ATP(-)**	**ATP(+)**	**ATP(-)**	**ATP(+)**	**ATP(-)**	**ATP(+)**	**ATP(-)**	**ATP(+)**
**H(r)**	0.225	0.224	0.233	0.225	0.230	0.219	0.215	0.218
**H(d)**	0.212	0.212	0.214	0.221	0.223	0.217	0.203	0.203
**S(r)**	0.095	0.066	0.082	0.095	0.087	0.095	0.113	0.108
**S(d)**	0.140	0.131	0.131	0.134	0.132	0.137	0.143	0.142
**Trn**	0.136	0.137	0.108	0.131	0.124	0.140	0.140	0.133
**Unrd**	0.192	0.236	0.229	0.200	0.200	0.192	0.187	0.199

Table S1. Secondary Structure Composition from Circular Dichroism Spectra. Numbers represent the fraction of each type of secondary structure, calculated as described in Supplementary Materials and Methods. H(r) denotes regular α-helix; H(d) denotes distorted α-helix; S(r) denotes regular β-sheet; S(d) denotes distorted β-sheet; Trn denote turn; Unrd denotes unoerdered.

Supplemental Figure Legends

Figure S1. Relative B-factors in the core (RecA homology) domain of human RAD51, calculated using PyMOL and PDB ID no. 1N0W. Blue denotes the lowest B-factors and red the highest. The positions of residues Asp-149, Arg-150, and Gly-151 are indicated on the structure. In general the domain is highly stable and the region in which tumor-associated mutations occur is well-ordered.

Figure S2. Circular dichroism spectra (mean residue ellipticity) of RAD51 wild-type (*red*), D149N (*green*), R150Q (*blue*), and G151D (*pink*) variants in (A) the absence and (B) the presence of 1 mM ATP. (C) Molar ellipticity of each variant at 222 nm, monitored as a function of temperature as an indicator of relative protein thermal stability.

Figure S3. SDS-PAGE of partial trypsinolysis reactions containing: *Lanes 1-4,* RAD51 wild-type in the presence of no nucleotide, 1 mM ATP, 1 mM ADP, and 1 mM ATPγS, respectively; *Lanes 5-8,* D149N in the presence of no nucleotide, 1 mM ATP, 1 mM ADP, and 1 mM ATPγS, respectively; *Lanes 9-12,* R150Q in the presence of no nucleotide, 1 mM ATP, 1 mM ADP, and 1 mM ATPγS, respectively; *Lanes 13-16,* in the presence of no nucleotide, 1 mM ATP, 1 mM ADP, and 1 mM ATPγS, respectively.

Figure S4. Electrophoretic mobility shift of RAD51 nucleoprotein complexes as a function of protein concentration and nucleotide. (A-C) Titrations of circular φX174 ssDNA (20 μM) with wild-type and variant RAD51 proteins (0-8 μM) in the presence of (A) no

nucleotide, (B) 1 mM ADP, and (C) 1 mM ATP. (D-F) Titrations of linear φX174 dsDNA (20 μM) with wild-type and variant RAD51 proteins (0-8 μM) in the presence of (A) no nucleotide, (B) 1 mM ADP, and (C) 1 mM ATP.

Figure S5. Yeast two-hybrid detection of wild-type (WT) RAD51 protein-protein interactions with itself and with variants D149N, R150Q, and G151D. Blue streaks are positive for protein-protein interactions. Bait/prey = (A) WT/WT; (B) D149N/WT; (C) R150Q/WT; (D) G151D/WT; (E) bait vector control/prey vector control; (F) WT/prey vector control; (G) bait vector control/WT. Reverse assays with WT as bait and D149N, R150Q, or G151D as prey were all positive (data not shown). All control assays with variants as bait or prey against the opposite empty vector were negative (data not shown).

Figure S6. Position of the RAD51 Schellman loop motif (*red*), with Arg-150 indicated, relative to the positions of bound BRC4 peptide from BRCA2 (*purple*), the binding region for PALB2 (*green*), and the putative binding region for p53 (*yellow*). The side chain of Arg-150 forms a hydrogen bond with the backbone at residue Tyr-178 within the putative p53 binding region. Structure rendered using PyMOL and PDB ID no. 1N0W.

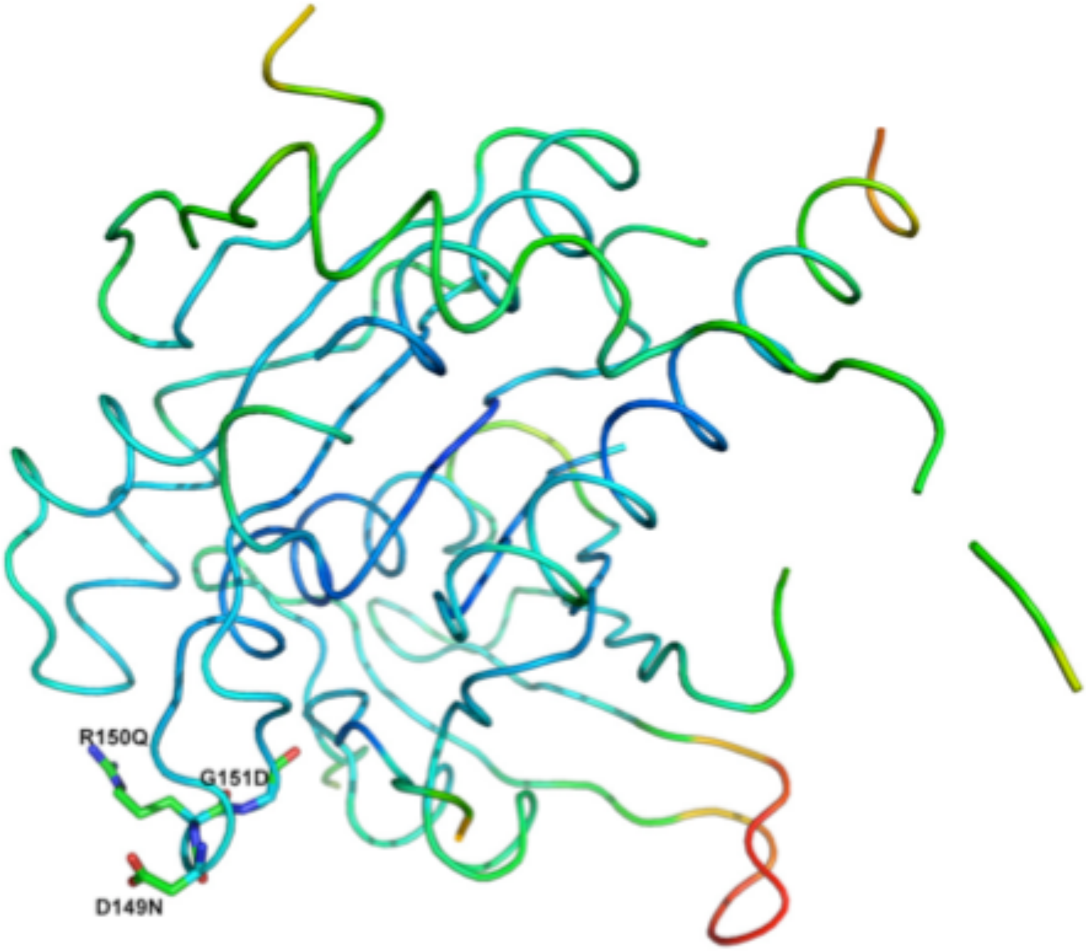

Figure S1

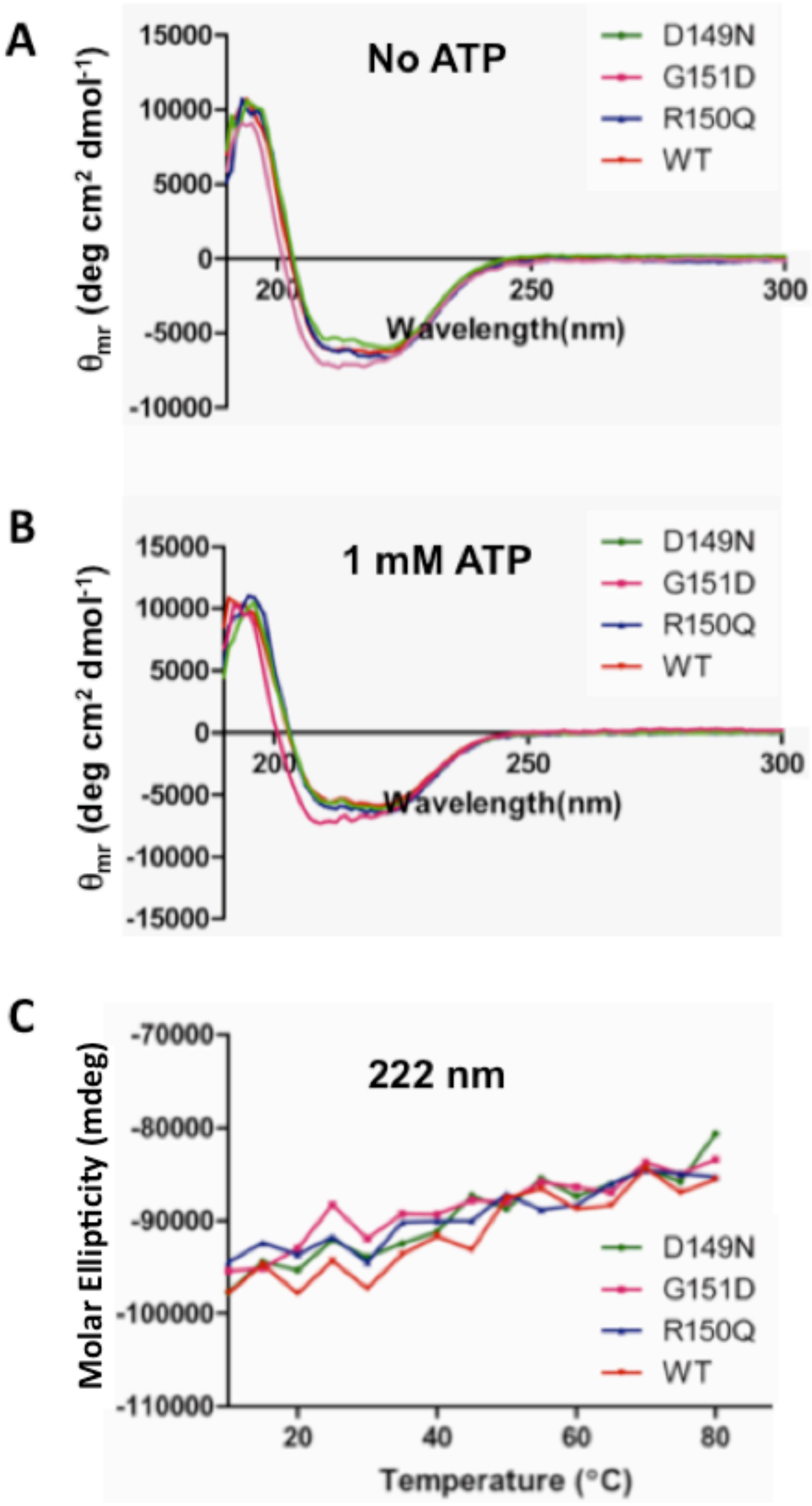
A
No ATP
D149N
G151D
R150Q
WT
15000
10000
5000
0
-5000
-10000
200
250
300
Wavelength(nm)
θ_{mr} (deg cm² dmol⁻¹)
B
1 mM ATP
D149N
G151D
R150Q
WT
15000
10000
5000
0
-5000
-10000
-15000
200
250
300
Wavelength(nm)
θ_{mr} (deg cm² dmol⁻¹)
C
222 nm
-70000
-80000
-90000
-100000
-110000
20
40
60
80
Temperature (°C)
Molar Ellipticity (mdeg)
D149N
G151D
R150Q
WT

Figure S2

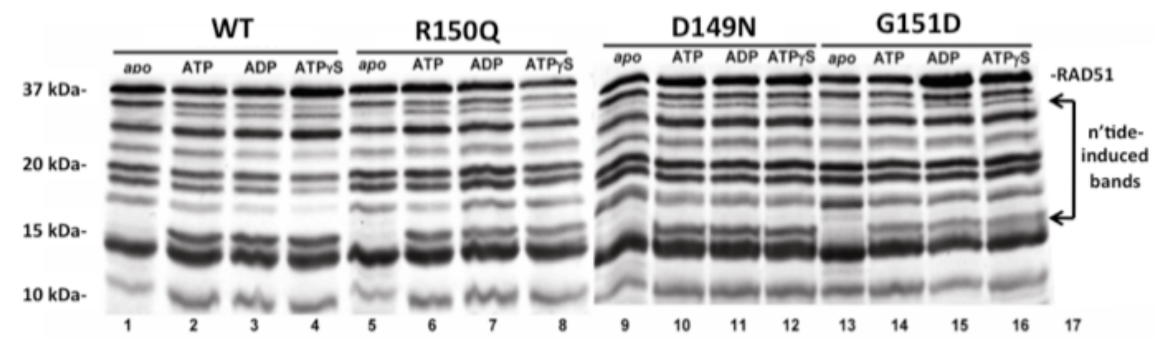

WT
R150Q
D149N
G151D
apo
ATP
ADP
ATPγS
37 kDa-
20 kDa-
15 kDa-
10 kDa-
-RAD51
n'tide-induced bands
1 2 3 4 5 6 7 8 9 10 11 12 13 14 15 16 17

Figure S3

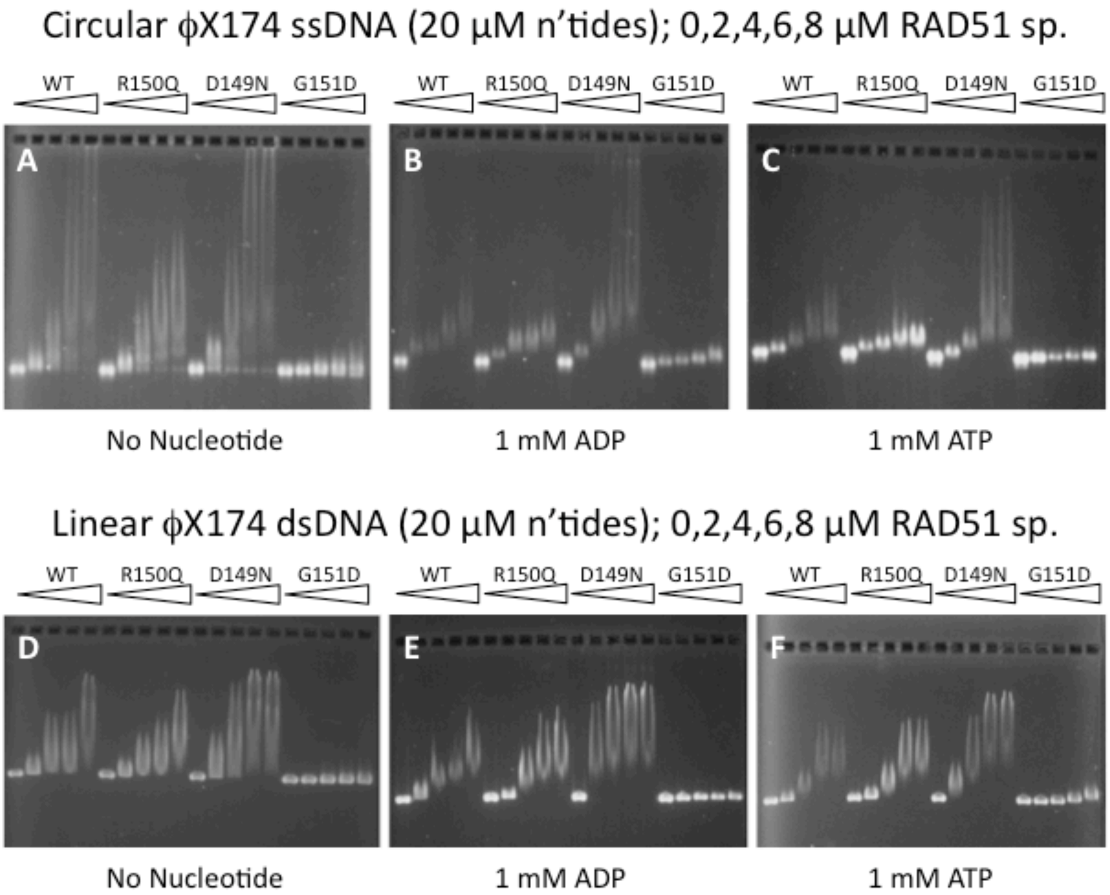
Circular ϕX174 ssDNA (20 μM n'tides); 0,2,4,6,8 μM RAD51 sp.
WT
R150Q
D149N
G151D
A
B
C
No Nucleotide
1 mM ADP
1 mM ATP
Linear ϕX174 dsDNA (20 μM n'tides); 0,2,4,6,8 μM RAD51 sp.
D
E
F

Figure S4

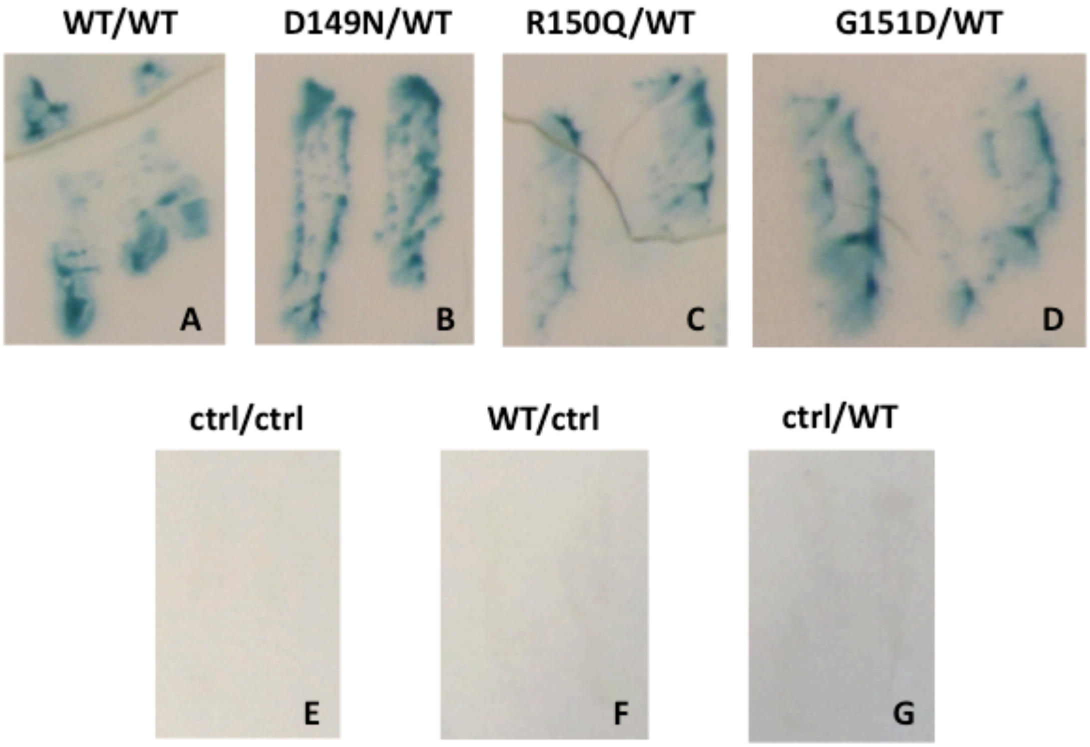
WT/WT
D149N/WT
R150Q/WT
G151D/WT
A
B
C
D
ctrl/ctrl
WT/ctrl
ctrl/WT
E
F
G

Figure S5

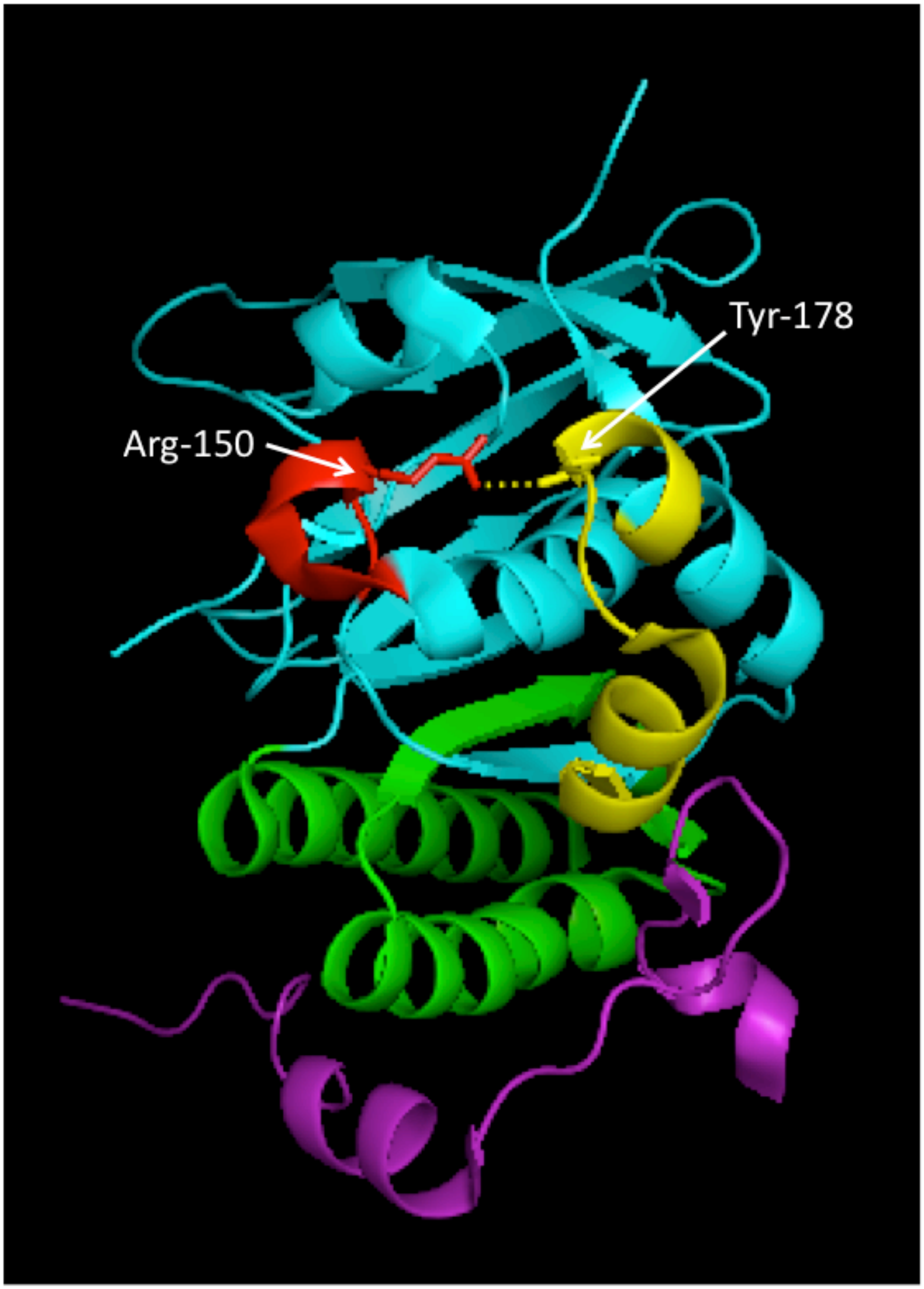

Figure S6
